# *Lactobacillus gasseri* LA806 Supplementation in Patients with Irritable Bowel Syndrome: A Multicenter Study

**DOI:** 10.3390/jcm11247446

**Published:** 2022-12-15

**Authors:** Samira Ait Abdellah, Julien Scanzi, Caroline Gal, Marc Martin, Marc Beck, Veronica Ojetti

**Affiliations:** 1 PiLeJe Laboratoire, 75015 Paris, France; 2 Centre Hospitalier de Thiers, 63300 Thiers, France; 3 UMR INSERM 1107, Neuro-Dol, Faculty of Medicine, Clermont-Auvergne University, 63000 Clermont-Ferrand, France; 4 Cabinet Médical, 76130 Mont-Saint-Aignan, France; 5 Cabinet Médical, 31240 L’Union, France; 6 Emergency Medicine Department, Fondazione Policlinico Universitario A. Gemelli, IRCCS, Catholic University, 00165 Rome, Italy

**Keywords:** microbiota, probiotics, irritable bowel syndrome, functional disease, abdominal pain, quality of life

## Abstract

The potential benefits of *Lactobacillus gasseri* LA806 in IBS were previously identified in a comprehensive preclinical research program. The purpose of this multicenter study was to explore in real-life conditions changes in IBS symptoms and quality of life in patients receiving a 4-week supplementation with *L. gasseri* LA806. Altogether 119 patients meeting Rome IV criteria for IBS were included, of whom 118 received the supplement. The majority of patients (71.8% (95% CI 63.6−79.9%)) manifested a ≥30% decrease in abdominal pain at 4 weeks, the mean abdominal pain score diminishing by 54.2% (from 5.3 ± 2.2 to 2.2 ± 2.4, *p* < 0.0001). A statistically significant decrease in abdominal pain was seen as early as the first week. A decrease of ≥30% in both abdominal pain score and global IBS symptom score was attained in 61.5% of patients (95% CI 51.7−71.2%). The mean IBS-SSS score fell by 152 ± 112 points (*p* = 0.001), with symptoms being attenuated in 85% of patients (CGI-I). Supplementation led to a 10-fold decrease in the number of patients reporting severe IBS symptoms. The concomitant intake of antidiarrheals, antispasmodics and analgesics decreased and quality of life scores significantly improved. These preliminary results warrant confirmation by a randomized, placebo-controlled study that this study will allow a better design.

## 1. Introduction

Irritable bowel syndrome (IBS) is one of the most frequent functional gastrointestinal disorders encountered by primary physicians and gastroenterologists [1]. The worldwide prevalence of IBS is estimated at 3–11%, with a wide variability across geographic areas, and depending on the Rome criteria used to define the condition [2,3,4,5]. The use of the Rome IV diagnostic criteria reduces the observed prevalence by approximately half, largely owing to the mandatory presence of abdominal pain, with similar values (4.4% to 4.8%) being reported in the United States, Canada and the United Kingdom [6]. The risk of IBS diagnosis is slightly higher in women than in men and in those aged ≤ 50 years [3,4,7].

IBS is characterized by chronic or recurrent abdominal pain associated with altered bowel habits, and sometimes with other symptoms such as bloating. According to Rome IV criteria, abdominal pain is a defining characteristic of IBS irrespective of its subtype (diarrhea-predominant [IBS-D], constipation-predominant [IBS-C], mixed [IBS-M] or unclassified [IBS-U]), independently impacting health-related quality of life and patient-reported disease severity [3,8,9]. Abdominal pain is recognized as the major symptom prompting IBS patients to seek medical advice [1] and the reduction in abdominal pain intensity is recommended as a primary endpoint in Food and Drug Administration (FDA) guidance for the clinical evaluation of treatments for IBS [10]. Overall, IBS adversely affects the quality of life, work productivity and social activities and may be associated with comorbid conditions such as anxiety, stress and depression [11,12].

The pathogenesis of IBS is multifactorial and remains only partially understood. However, the accumulating research data suggest that alterations in the gut microbiota balance, increased permeability of the intestinal epithelial barrier, activation of nociceptive sensory pathways and dysregulation of the enteric nervous system play important roles [13,14,15,16,17,18,19]. Such phenomena lead to a breakdown of tolerance to the gut microbiome and to an inappropriate and persistent inflammatory response.

These findings have encouraged the evaluation of probiotics, principally the *Lactobacillus* and *Bifidobacterium* species, for the management of IBS. Recent meta-analyses indicate a significant beneficial effect of probiotics on IBS symptoms as a whole, as well as on the abdominal pain score and quality of life, without significant side effects compared to placebo [20,21,22,23]. Consistent with the lower abundance of *Lactobacillus* and *Bifidobacterium* species observed in IBS patients, particularly in those with more severe disease [24], randomized, placebo-controlled trials and observational studies have shown a reduction in IBS symptom severity [1,25,26,27,28,29,30,31] and improved quality of life [25,26,27,29,30,31] following supplementation with probiotic strains from these genera, including *L. gasseri* [26,32].

The potential benefits of *L. gasseri* LA806 were demonstrated in a comprehensive preclinical research program focusing on the pathophysiology of IBS, including both in vitro and in vivo studies. In particular, *L. gasseri* LA806 adhered strongly to intestinal epithelial cells and restored and reinforced the epithelial barrier in an in vitro model comprising Caco-2 cell monolayers sensitized with hydrogen peroxide to induce epithelial barrier permeability [16]. In a rat model of IBS pathophysiology, *L. gasseri* LA806 significantly reduced both hyperpermeability of the intestinal epithelial barrier and visceral hypersensitivity induced by chronic stress [33]. Adhesion of live *L. gasseri* LA806 to bovine mammary epithelial cells (bMECs) induced a five-fold decrease in *Staphylococcus aureus* adhesion and internalization as well as reducing pro-inflammatory cytokine expression by *S. aureus*-stimulated bMECs [34]. In a study in *Candida albicans*-infected mice, *L. gasseri* LA806 alone, or combined with *L. helveticus* LA401, decreased *Candida* colonization of the esophageal and gastrointestinal tract in mice, significantly shifting the composition of the gut microbiota towards a protective profile and reducing colon inflammation and oxidative stress [35].

The aim of this study was to explore in real-life conditions changes in IBS symptoms and quality of life in patients affected by IBS according to Rome IV criteria (irrespective of subtype) after 4-week supplementation with *L. gasseri* LA806.

## 2. Materials and Methods

### 2.1. Study Design

Patients with IBS according to Rome IV criteria and meeting the inclusion criteria and none of the exclusion criteria were invited to participate in the study during consultations with office-based primary physicians and gastroenterologists in 25 centers throughout France. The study protocol (no. 2019-A01141-56) and the patient information sheet were approved by the local Ethics Committee (Comité de Protection des Personnes Ile de France 1 IRB / IORG number: IORG0009918) on 11 June 2019. In accordance with the commitment to comply with relevant legislation and regulations, a declaration of conformity was addressed to the French national commission for personal data privacy (Commission Nationale de l’Informatique et des Libertés [CNIL]). The study was registered on the ClinicalTrials.gov site (accessed on 4 October 2022): registration number NCT04324658. It was conducted according to the Declaration of Helsinki and the Strengthening the Reporting of Observational Studies in Epidemiology (STROBE) guidelines.

The study comprised two visits, one for inclusion (V1) and one at the end of the 4-week supplementation phase ± 2 days (V2), based on a routine practice follow-up. A box containing 56 capsules, each containing 5 × 10^9^ colony-forming units (CFU) of *L. gasseri* LA806 with corn starch and magnesium stearate as excipients (Lactiplus, PiLeJe Laboratoire), was given to each patient enrolled by the investigators at the end of V1. Patients were instructed to swallow 2 capsules per day with a glass of water 20 min before a meal from day (D)1 to D28.

### 2.2. Patient Cohort

The cohort of patients consisted of male and female adults aged ≥18 years suffering from IBS according to Rome IV criteria [36] irrespective of subtype. Additional inclusion criteria comprised symptom onset at least 6 months before diagnosis, the presence of recurrent abdominal pain on an average of 1 day a week during the last 3 months and access to a computer or tablet with an internet connection allowing completion of the online questionnaire. Non-inclusion criteria comprised the presence of clinical signs of alarm such as rectal bleeding, fever, suspicion or evidence of unexplained weight loss, suspicion or evidence of diseases excluding the diagnosis of IBS including (but not limited to) chronic inflammatory bowel disease (Crohn’s disease, ulcerative colitis), colorectal cancer, celiac disease, hyperthyroidism, intestinal infection, lactose intolerance, other intolerance, bile acid malabsorption and other malabsorption syndromes (e.g., fructose), a history of abdominal surgery other than appendectomy, allergy or hypersensitivity to any of the ingredients of the supplement, inability to understand the study information sheet or complete the online questionnaire, and inclusion in another clinical study. Patients continued to take their usual treatments.

All patients eligible for inclusion received written information about the study and gave their verbal consent to participate prior to their enrollment in compliance with French regulations for observational studies. Participants were informed that they could voluntarily discontinue the study at any time without prejudice. Participants did not receive remuneration.

### 2.3. Data Collection

**Figure 1:**
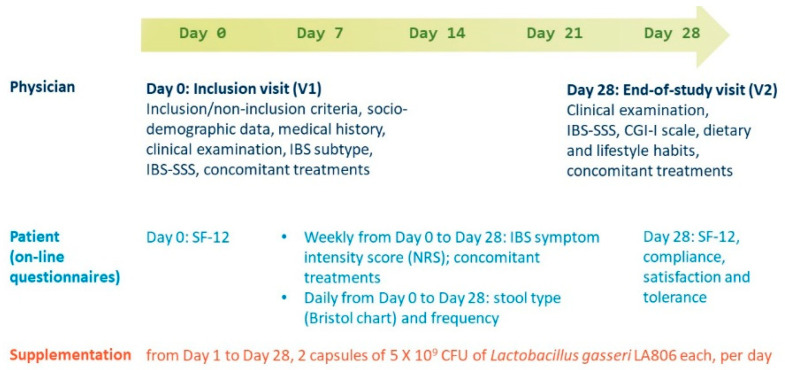
Study design. IBS-SSS, Irritable Bowel Syndrome Symptom Severity Scale; CGI-I scale, Clinical Global Impression of Improvement scale; SF-12, 12-item Short Form survey (quality of life questionnaire); V, visit; NRS, numeric rating scale.

Data were collected by the investigators at the inclusion visit (V1) and the end-of-study visit (V2) as well as by the patients in online questionnaires completed on a daily and weekly basis during supplementation at home (Figure 1).

At V1, IBS subtypes were defined according to the Bristol classification [3,37] based on the predominant nature of the abnormal bowel movements, as constipation (IBS-C), diarrhea (IBS-D), mixed (both constipation and diarrhea; IBS-M) or unclassified (IBS-U), symptom severity was rated according to the IBS Symptom Severity Scale (IBS-SSS) questionnaire [38] and concomitant treatments were recorded. At V2, global change since V1 was assessed according to the Clinical Global Impression of Improvement (CGI-I) scale, the IBS-SSS questionnaire was completed and concomitant treatments were recorded.

Patients completed a quality of life questionnaire (12-item Short Form survey [SF 12]) at D0 and D28, recording IBS symptom intensity (numeric rating scale [NRS]) and concomitant treatments on a weekly basis and stool type (Bristol Stool Form Scale [BSFS]) and frequency every day, from D0 to D28.

### 2.4. Evaluation Criteria

#### 2.4.1. Primary Endpoint

The response to a 4-week supplementation with the probiotic in terms of abdominal pain (item 1b on the IBS-SSS questionnaire [38]) was expressed as the proportion of patients showing an improvement in abdominal pain score of at least 30%, on a 10-point NRS, between inclusion (V1) and the end of the 4-week supplementation period (V2) [10].

#### 2.4.2. Secondary Endpoints

- Change in abdominal pain score (item 1b in the IBS-SSS questionnaire) on a 10-point NRS between V1 and V2;
- Rate of response to supplementation defined as an improvement of at least 30% on a 10-point NRS in both global IBS symptom intensity score and abdominal pain score between V1 and V2;
- Change relative to baseline values of individual IBS symptom intensity scores, global IBS symptom intensity score, IBS-SSS total score and stool frequency during supplementation;
- Proportions of patients experiencing, respectively, no change, worsening and improvement in symptom intensity according to their IBS-SSS score class at V1 and V2;
- Proportions of patients experiencing during supplementation no change in predominant stool pattern, a trend towards more solid stools and a trend towards more liquid stools, respectively, according to the BSFS;
- Physician’s subjective assessment of the patient’s symptoms as a whole, according to the CGI-I scale (very much improved, much improved, minimally improved, no change, minimally worse, much worse or very much worse) between V1 and V2;
- Change in individual quality of life scores (SF-12) from inclusion to the end of the 4-week supplementation period;
- Change in the mean weekly number of concomitant treatments taken to relieve IBS symptoms during the study;
- Rate and type of adverse events experienced during the study.

### 2.5. Statistical Analyses

All analyses were performed using SAS software version 9.4 (SAS Institute Inc., Cary, NC, USA) in accordance with ICH-E9 guidelines. The analyses were performed on the three standard statistical populations: the safety population, including all patients enrolled and having received the study supplementation, the intention to treat (ITT) population comprising all patients having received the study supplementation and having undergone the assessment of primary criterion, and the per protocol (PP) population comprising all patients having completed the study as planned with no deviations from the protocol. Quantitative parameters were described in terms of the number of patients, mean, standard deviation (SD), median, first and third quartiles, and minimum and maximum values. Qualitative parameters were described as frequencies and percentages. The two-sided alpha risk was set at 5%.

The rate of responders is presented with its 95% confidence interval (CI). With regard to secondary endpoints, Chi-square tests were used to compare subgroups for categorical variables, Student’s *t*-tests and analysis of variance (ANOVA) tests with post hoc comparisons being used to compare mean scores for continuous variables. Correlations between variables were assessed by Spearman correlation coefficients. Weekly changes in symptom scores, stool frequency and the number of concomitant medications taken during the supplementation period were analyzed by ANOVA for repeated measures. Quality of life (SF-12) scores and summary measures were compared between inclusion and the end of the 4-week supplementation period using paired Student’s *t*-tests. Changes in stool consistency and intake of concomitant treatments during supplementation were analyzed by Cochran Armitage tests and McNemar tests. For analysis of the change in abdominal pain intensity between inclusion and the end of supplementation at 4 weeks, based on the numbers and percentages of patients experiencing abdominal pain considered as absent to mild, moderate and severe, respectively, at the two timepoints, the symmetry of distribution was checked by means of an intragroup test. Missing data were not replaced.

#### 2.5.1. Analysis of Prognostic Factors

Univariate logistic regressions were performed to investigate potential prognostic factors with regard to the supplementation response among the following parameters: IBS subtype, age, sex, body mass index (BMI), time since diagnosis, comorbidities, quality of life (SF-12), concomitant treatments (including antidiarrheals, antispasmodics, laxatives, antibiotics, probiotics, prebiotics, paracetamol, aspirin, ibuprofen and other NSAIDs), lifestyle and diet changes and compliance. Multivariate logistic regression was performed for the parameters with a *p*-value < 0.20 using an automatic stepwise process of parameter selection based on a statistical significance threshold of *p* < 0.05.

#### 2.5.2. Calculation of Sample Size

The required sample size was calculated on the basis of clinical trial data and the estimated response rate. Data from randomized, controlled trials evaluating probiotics indicate that about 25% of patients receiving a placebo and 45% of those receiving probiotics respond to treatment in terms of abdominal pain relief [1,39]. Assuming a response rate for the primary endpoint of 45%, a 5% two-sided alpha risk and a 90% power, it was calculated that 95 patients would need to be included in the study in order to describe the response rate with a precision of 10%. Based on an estimated 20% rate of patients lost to follow-up or with missing data, the planned study population comprised 119 patients.

## 3. Results

The study was conducted between July 2019 and June 2020. The first patient was included on 30 July 2019 and the last visit of the last patient took place on 26 June 2020.

### 3.1. Patient Cohort

**Figure 2:**
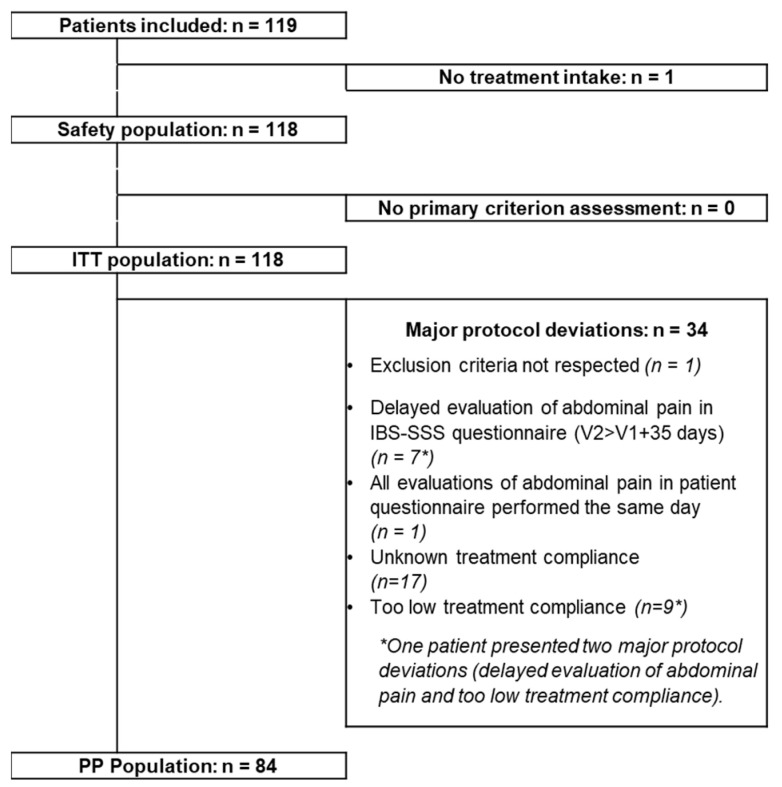
Flow chart of patient populations. ITT, intention to treat; PP, per protocol; V, visit; IBS-SSS, Irritable Bowel Syndrome Symptom Severity Scale.

In total 119 patients were included in the study (Figure 2). However, one patient discontinued the study two days after inclusion, due to a lung infection, without having taken the probiotic. The ITT population, having completed the evaluation of the primary endpoint as planned, comprised 118 patients (24 men and 94 women), the PP population comprises 84 patients. The results concerning the effects of supplementation described below are based on analyses of the ITT population. The 118 patients attended V2. The principal characteristics of this population at inclusion in terms of demographics, time since diagnosis of IBS, IBS subtype and severity of IBS are presented in Table 1.

### 3.2. Decrease in Abdominal Pain Score

The analysis of the primary endpoint, defined as the rate of patients presenting an improvement of at least 30% in abdominal pain score (item 1b on the IBS-SSS questionnaire) between V1 (inclusion) and V2 (after 4 weeks of supplementation), showed that this target was attained by 71.8% of patients (95% CI 63.6−79.9%). After univariate logistic regression, no relationship was observed between the achievement of the primary endpoint and patient age, sex, BMI, time since diagnosis, compliance with supplementation, IBS subtype, IBS severity, concomitant treatments, main comorbidities or changes in lifestyle or dietary habits during the study.

### 3.3. Change in Abdominal Pain Score

The mean score for abdominal pain (item 1b on the IBS-SSS questionnaire) decreased by 54.2% (*p* < 0.001) between inclusion and the end of the 4-week supplementation period (5.3 ± 2.0 and 2.2 ± 2.4, respectively; *p* < 0.001), corresponding to a reduction in pain severity from moderate to mild. The changes in abdominal pain intensity from inclusion to the end of the supplementation period expressed as the number and percentage of patients considering this pain to be absent or mild (scores 0–3 on a 10-point scale), moderate (scores 4–6) and severe (scores 7–10), respectively, are shown in Table 2. The proportion of patients recording abdominal pain intensity as moderate or severe decreased from 80% at inclusion to 28% at the end of 4 weeks of supplementation, with 72% experiencing no or mild pain at that time point. The percentage of patients presenting severe pain decreased by a factor of 4. A significant decrease in abdominal pain was seen by the end of the first week of supplementation (*p* < 0.0001).

### 3.4. Decrease in Both Abdominal Pain Score and Global IBS Symptom Intensity Score

A decrease of at least 30% in both abdominal pain score and global IBS symptom intensity score (assessed by the patients on NRS) was achieved by 61.5% of patients (95% CI 51.7–71.2%). The statistical analysis of the relationship between this response to supplementation and various potential prognostic factors showed no significant relationship with age, sex, BMI, time since diagnosis, compliance with treatment, IBS subtype, IBS severity, main comorbidities, changes in lifestyle habits or diet during the study or concomitant treatments. In contrast, a statistically significant relationship was observed with several quality of life (SF-12) scores, namely role physical functioning score (*p* = 0.014), role emotional functioning score (*p* = 0.031), social functioning score (*p* = 0.005), mental health score (*p* = 0.008), general health score (*p* = 0.013), physical health composite score (PCS; *p* = 0.025) and mental health composite score (MCS; *p* = 0.005), responders showing higher mean values for all these scores.

### 3.5. Changes in IBS Symptom Intensity Score

**Figure 3:**
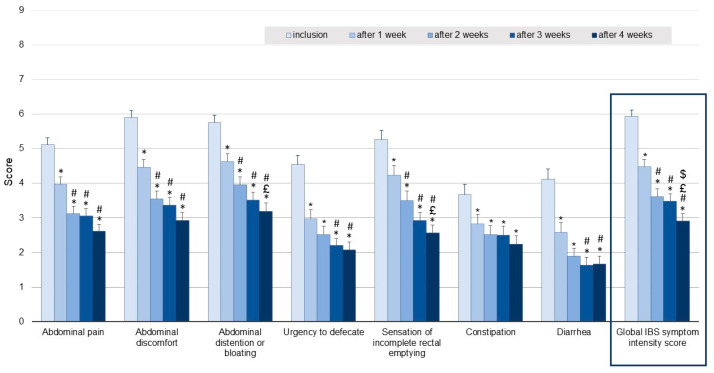
Week-by-week changes in individual and global (box) IBS symptom intensity scores from inclusion to the end of the 4-week supplementation period (mean ± SEM). Assessed by the patients on numeric rating scales (NRS). * *p* < 0.05 compared to inclusion; # *p* < 0.05 compared to week 1; £ *p* < 0.05 compared to week 2; $ *p* < 0.05 compared to week 3.

Comparison of the global IBS symptom intensity score, including abdominal pain, abdominal discomfort, abdominal distension or bloating, urgency to defecate, sensation of incomplete rectal emptying, constipation and diarrhea (assessed by the patients on NRS), reported for the 7-day period preceding inclusion with that recorded for the final week of supplementation (week 4) revealed a decrease of 49.1%, from 6.1 ± 1.7 to 2.9 ± 2.1 (*p* < 0.001) (Figure 3). Significant decreases in mean global IBS symptom intensity scores were seen after one and two weeks of supplementation, the mean score then remained stable during the last two weeks of the study.

Similar to the improvement in the global IBS symptom intensity score, most of the improvements in individual symptoms shown in Figure 3 occurred after only one week of supplementation, these improvements being maintained during the following three weeks.

### 3.6. Changes in IBS Symptom Severity Scale Score

**Figure 4:**
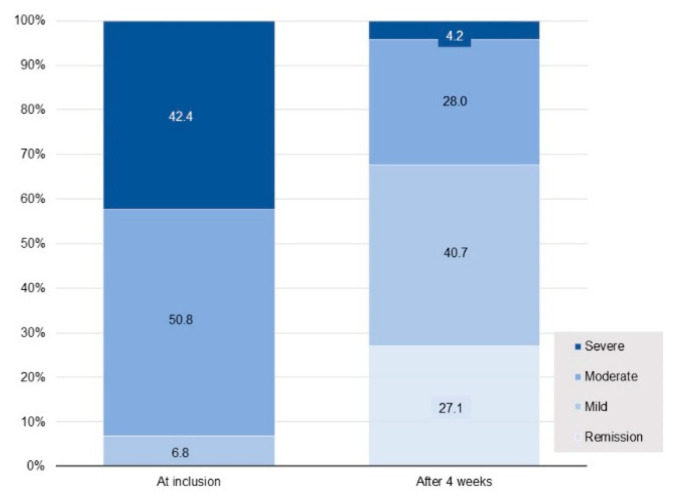
Evolution of IBS severity between inclusion and the end of the 4th week of supplementation. Percentages of patients with IBS rated as severe (IBS-SSS score: [300–500]), moderate (IBS-SSS score: [175–299]), or mild (IBS-SSS score: [75–174]) and percentage of healthy patients (IBS-SSS score: <75, corresponding to remission) at inclusion and after 4 weeks of supplementation. Patient distribution between IBS-SSS classes at 4 weeks differed to a statistically significant extent from that observed at inclusion (*p* < 0.0001).

In the ITT population, mean (± SD) IBS-SSS score decreased from 291.8 ± 78.3 at inclusion to 139.8 ± 98.4 at the end of the 4-week supplementation period, corresponding to a substantial mean decrease of 152 ± 112 points (*p* = 0.001). In total, 81.4% of patients manifested a clinically relevant decrease of at least 50 points in the IBS-SSS score, 64.4% presented a decrease of at least 95 points. No relationship was seen between the mean change in this score from inclusion to the 4-week assessment and age, sex, BMI, time since diagnosis, IBS subtype, main comorbidities (with the exception of the MedDRA System Organ Class “Musculoskeletal disorders and connective disorders”), changes in lifestyle or dietary habits during the study or concomitant treatments. In contrast, statistically significant differences (*p* < 0.05) were observed in relation to IBS severity (patients with severe disease showing a greater score improvement) and between patients with (*n* = 19) and without (*n* = 99) musculoskeletal disorders, such as fibromyalgia and osteoarthritis (the decrease in IBS score being smaller in patients with these disorders). Overall, IBS-SSS at 4 weeks indicated IBS remission in 27% of patients, improvement in 50%, no change in 20% and worsening of symptoms in 3%. The percentage of patients suffering from severe IBS showed a 10-fold decrease, falling from 42.4% at inclusion to only 4.2% after 4 weeks of supplementation (Figure 4). The patient distribution between the different IBS-SSS classes at 4 weeks differed to a statistically significant extent from that observed at inclusion (*p* < 0.0001).

### 3.7. Changes in Stool Pattern

In the ITT population, statistically significant improvements in stool consistency were observed in patients with IBS-D between week 1 and week 4, with a significant decrease in a mean score of −1.47 ± 1.42 on the BSFS (from 5.4 ± 1.02 at week 1 to 3.92 ± 1.23 at week 4, *p* < 0.05). No statistically significant changes were noted in patients with IBS types C, M or U.

No statistically significant differences between weekly stool frequencies were seen during the supplementation period either in the ITT population as a whole or in patients with IBS types C, D, M or U, respectively. In contrast, analysis of the PP population showed a statistically significant increase in stool frequency in patients with IBS-C during the study (two-way ANOVA for repeated measures *p* < 0.05). The mean weekly number of stools increased by +1.73 ± 3.52, from 6.86 ± 2.88 at week 1 to 8.59 ± 4.47 at week 4.

### 3.8. Changes in Quality-of-Life Scores

Analyses of SF-12 scores after 4 weeks of supplementation compared to those measured at inclusion showed significant increases in overall score (6.2 ± 10.7 points, *p* < 0.001), MCS (4.2 ± 9.7 points, *p* < 0.001) and PCS (6.2 ± 10.7 points, *p* < 0.001), as well as in several individual domain scores. Statistically significant increases in mean scores for role physical functioning, role emotional functioning, social functioning, mental health, bodily pain (all *p* < 0.001) and general health (*p* < 0.006) were also observed.

### 3.9. Physician’s Clinical Global Impression of Improvement (CGI-I)

Overall, 85% of patients experienced an improvement in IBS during the study period according to physician assessments using the CGI-I scale. At the end of 4 weeks of supplementation, 20% of patients were considered to be “very much improved”, 37% to be “much improved”, 28% to be “minimally improved”, 9% to have experienced “no change”, 4% to be “minimally worse” and 2% to be “much worse”. In total, 88% of the patients declared that their state of health had improved and 81% expressed satisfaction with the probiotic supplement taken.

### 3.10. Changes in Concomitant Treatment Intake

**Figure 5:**
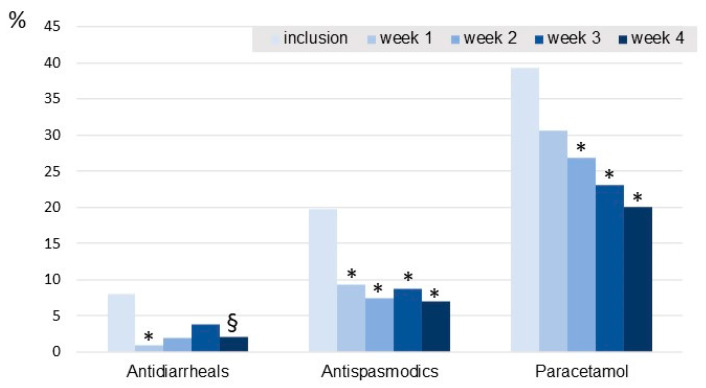
Changes in concomitant treatment intake from inclusion to the end of the 4-week supplementation period expressed as the percentage of patients treated at each time point. * *p* < 0.05 compare to the percentage treated at inclusion; § *p* = 0.0588.

The percentage of patients taking aspirin, ibuprofen or other NSAIDs tended to decrease over the course of the study (significant difference observed between inclusion and week 1). The intake of antidiarrheals decreased during the 4 weeks of supplementation, the decrease being significant in the first week (Figure 5). Intakes of paracetamol and antispasmodic drugs decreased significantly during the 4-week supplementation.

### 3.11. Compliance

Mean compliance, expressed as the number of capsules actually consumed relative to the theoretical number according to the study protocol (100 × no. of capsules consumed/no. of days of intake × daily dose), was 96.1 ± 13.2% (median value: 100%). Mean ± SD exposure to the supplementation was 27.3 ± 2.2 days with a median of 28.0 days and a range of 14.0–28.0 days (Q1-Q3: 28.0–28.0).

### 3.12. Satisfaction

The majority of patients (88.2%) considered that the supplementation helped to improve their health, 81.2% declared that they were satisfied with the probiotics tested, 72.3% that they would like to continue taking it and 81.2% that they would recommend it.

### 3.13. Tolerance

No serious adverse events were observed during the supplementation. A total of 14 treatment-emergent adverse events (TEAE) were reported, of which 6 were considered to be related to the supplementation: pain in 2 patients, abdominal distension, constipation, rectal bleeding and dysuria in 1 patient in each case. Four TEAE led to the discontinuation of the study supplementation: pain in two patients, abdominal distension in one patient, and malaise in one patient. Overall, the probiotic was very well tolerated by 88.1% of the ITT population and moderately well tolerated by a further 10%.

## 4. Discussion

This multicenter study collected and analyzed data on symptoms and quality of life in real-life conditions in patients affected by IBS supplemented for 4 weeks with *L. gasseri* LA806, previously identified for its properties in a preclinical research program. We observed a decrease in abdominal pain and IBS symptom severity, and improvement in the quality of life, stool frequency and stool consistency in IBS patients.

The percentage of patients reporting severe symptoms of IBS in the IBS-SSS questionnaire decreased by a factor of 10 after 4 weeks of supplementation with *L. gasseri* LA806, falling from 42.4% at inclusion to 4.2% at the end of the study. The mean IBS-SSS score decreased by over 50% from inclusion to the end of the 4-week supplementation period, corresponding to a mean fall of 152 points, exceeding by a factor of three the minimum decrease of 50 points considered to indicate a clinically relevant improvement [38]. These results compare favorably with those of other published studies on *Lactobacillus* or *Bifidobacterium* species. Pedersen et al. observed a decrease of 68 points in mean IBS-SSS score following a 6-week supplementation with *L. rhamnosus* GG [40], while Lewis et al. reported decreases of 50 and 75 points, respectively, after 4 and 8 weeks of supplementation with *L. paracasei* HA-196 (no difference with placebo) [29]. Martoni et al. reported decreases of 133 and 105 points in mean IBS-SSS scores following a 6-week supplementation with *L. acidophilus* DDS-1 and *Bifidobacterium lactis* UABla-12, respectively, the mean decreases of 56 and 50 points, respectively, being observed after 3 weeks of supplementation [30]. A decrease of 101 points in IBS-SSS score from inclusion to the end of an 8-week supplementation with heat-inactivated *B. bifidum* MIMBb75n was described by Andresen et al., 41% of patients receiving *B. bifidum* achieving an improvement in IBS-SSS score of at least 50% compared to 29% of those receiving placebo [41]. One obvious limitation of our study is the absence of a control group, especially since the placebo effect is important in IBS. However, we have just seen that a decrease of 150 points in the IBS-SSS score is far beyond a decrease linked to a placebo effect; in the studies cited above, the decrease observed in the placebo group was between 35 and 60 points. In our study, in addition to the 152-point decrease in mean IBS SSS score observed, 55.9% of patients achieved an improvement in IBS-SSS score of at least 50% within the 4-week supplementation period.

Abdominal pain is a key contributor to patient impressions of disease severity and a major factor impairing the health-related quality of life [8,20]. In our study, rapid relief of this symptom was observed, a statistically significant improvement being evident after only one week. By the end of the 4-week supplementation period, 72% of patients had achieved the primary endpoint, i.e., an improvement of at least 30% in IBS-SSS abdominal pain score compared to baseline. Martoni et al. reported a greater than 30% reduction in abdominal pain severity measured on the Abdominal Pain Severity—Numeric Rating Scale (APS-NRS) in 52% and 28% of patients after a 6-week supplementation with *L. acidophilus* DDS-1 and *B. lactis* UABla-12, respectively, compared to 16% of patients receiving placebo [30]. Ducrotté et al. observed a 52% decrease in the mean frequency of abdominal pain after 4-week supplementation with *L. plantarum* 299v compared with a 14% reduction in the placebo group, the mean decreases in abdominal pain severity score measured on a visual analogue scale being 45% and 23%, respectively [1]. However, the latter study involved patients from India, with mainly male IBS-D patients, and the effects reported might not be replicated in our western population. A significantly greater reduction in abdominal pain score compared to placebo was also seen after 4 weeks of supplementation with two capsules of 5 × 10^9^ CFU/day of *L. gasseri* BNR17 [32].

The proportion of patients recording abdominal pain intensity as moderate or severe decreased significantly from 80% at inclusion to 28% at week 4 irrespective of patient age, sex, concomitant treatments, or changes in lifestyle or dietary habits during the study period. Decreases of at least 30% in individual and global IBS symptom intensity scores were observed in the majority of patients after only 2 weeks of supplementation with *L. gasseri* LA806. A 30% decrease in both abdominal pain score and global IBS symptom intensity score was achieved by 61.5% of patients.

These changes were accompanied by statistically significant improvements in several health-related quality of life (SF-12) scores, an improvement similarly reported in previous placebo-controlled studies investigating the effects of *L. gasseri* (*L. gasseri* CP2305 [26]; breast milk-derived *L. gasseri* BNR17 [27]). In our study, responders to supplementation manifesting a decrease of at least 30% in both abdominal pain score and global IBS symptom intensity score showed statistically significant increases in role physical functioning, role emotional functioning, social functioning, mental health and general health quality-of-life scores, as well as in both PCS and MCS. An analysis of quality-of-life parameters in the entire patient population revealed significant increases in the scores for all the above parameters as well as in bodily pain score. The mean overall SF-12 score increased significantly by 6.2 points from inclusion to the end of the 4-week supplementation with *L. gasseri* LA806, the mean MCS score increasing by 4.2 points and the mean PCS by 2.0 points. These results were similar to those of a study assessing the effects of a longer (8-week) supplementation with heat-inactivated *B. bifidum* MIMBb75 showing increases of 5.8, 3.3 and 2.5 points, respectively, in mean SF-12 score, MCS and PCS [41].

Concerning stool frequency and consistency, we observed a statistically significant increase in mean stool frequency (+1.73) in patients with IBS-C, as well an improvement in stool consistency (decrease in mean score of 1.47) in patients with IBS-D. With respect to IBS-C, an increase of one point is clinically relevant according to FDA guidance for clinical evaluation of treatments for IBS [10]. Similar results have been observed in other studies [26,30,41]; for example, Andresen et al. reported a significant 1.7-fold increase in weekly stool frequency in patients with IBS-C and a significant decrease in BSFS stool consistency score of 0.68 following an 8-week supplementation with *B. bifidum* HI-MIMBb75 [41].

In accordance with the improvements observed in our study in terms of overall IBS severity, abdominal pain and quality of life, weekly intakes of antidiarrheals, antispasmodics and paracetamol decreased compared to baseline values throughout the 4-week supplementation period. Furthermore, the supplementation was well tolerated with few adverse events reported. It should also be noted that no patients were lost to follow-up during this study; all 118 patients included in the ITT population attended V2. Also, the quality of the data collected was excellent; the patients completed the questionnaires assiduously throughout the study (102 out of 113 questionnaires have been completed). In addition, the two-capsule dose was well accepted by the patients, with compliance being very good.

Another potential bias of this study related to its observational nature is the intended non-prohibition of concomitant treatments. However, this point was anticipated from the design of the study, and consequently, a prospective collection of all concomitant treatments was carried out and univariate and multivariate analyses were planned. These analyses showed that the use of concomitant treatments had no impact on the response to supplementation with *L. gasseri* LA806. Moreover, we wanted to evaluate the evolution of the intake of these treatments during complementation with the strain and observed that the intake of antispasmodics and paracetamol in particular decreased significantly during the study. No relationship was observed between the rate of achievement of the primary endpoint (decrease in abdominal pain score) and IBS subtype, concomitant treatments or changes in lifestyle or dietary habits during the study. The advantage of a real-life study, as the term indicates, is to evaluate a treatment or supplement under real-life conditions. This approach enabled the assessment of the value of the supplement tested in patients not selected according to strict inclusion and non-inclusion criteria, and therefore, it is more representative of the population for which use of this supplement might be advocated (with comorbidities, concomitant treatments, etc.). It also confirmed the dosage and its acceptability by the patients, permitted assessment of the impact of this supplement on the recourse to concomitant treatments, and estimation of the likely compliance with supplementation. In view of the results obtained, *L. gasseri* LA806 appears to warrant further evaluation in a randomized study against a placebo to definitively verify its efficacy under controlled conditions.

## 5. Conclusions

This multicenter study conducted by primary care physicians and office-based gastroenterologists, in real-life conditions, permitted the collection of preliminary data in IBS patients supplemented with a probiotic strain that has been shown to be useful in preclinical models of IBS. The results suggest that a 4-week supplementation with *L. gasseri* LA806 may reduce abdominal pain and improve the quality of life of patients with any form of IBS. We observed a 10-fold decrease in the number of patients reporting severe IBS symptoms, with a more than 50% decrease in the mean IBS-SSS score. At the end of the supplementation period, 85% of patients experienced an improvement in their disease, with the remission of overall symptom severity in 27% of patients. Moreover, compliance and satisfaction were high and the supplementation was well tolerated, with very few adverse events recorded. These results confirm the potentially beneficial properties of this probiotic strain suggested by an extensive preclinical research program and will facilitate the design of a randomized placebo-controlled study.

## Figures and Tables

**Table 1:** Baseline patient characteristics (ITT population, *n* = 118).

Variable	Value
Age, years	
Mean ± SD	46.3 ± 15.5
Median (range)	46 (18–84)
Q1–Q3	32–58
Sex, no. (%)	
Male	24 (20.3)
Female	94 (79.7)
BMI (kg/m^2^)	
Mean ± SD	24.3 ± 5.1
Median (range)	24 (44–104)
Q1–Q3	20–27
Time since diagnosis of IBS, years (*n* = 117)	
Mean ± SD	7.13 ± 8.80
Median (range)	4.10 (0.00–49.12)
Q1–Q3	1.35–9.31
IBS subtype, no. (%)	
Constipation ^1^	27 (22.9)
Diarrhea ^2^	36 (30.5)
Mixed ^3^	49 (41.5)
Unclassified	6 (5.1)
Severity of IBS (IBS-SSS score), no. (%)	
Mild (75 ≤ total score ≤ 175 points)	8 (6.8)
Moderate (175 < total score ≤ 300 points)	60 (50.8)
Severe (total score > 300 points)	50 (42.4)

BMI, body mass index; IBS, irritable bowel syndrome; IBS-SSS, IBS Symptom Severity Scale; Q, quartile; SD, standard deviation. ^1^ abnormal stools predominantly of type 1 or 2; ^2^ abnormal stools predominantly of type 6 or 7; ^3^ >25% of abnormal stools of constipation type and >25% of diarrhea type.

**Table 2:** Change in abdominal pain intensity scores from inclusion to the end of the 4-week supplementation period (IBS-SSS item 1b).

Abdominal Pain Intensity (IBS-SSS Score)	No. (%) of Patients (*n* = 118)	
	At Inclusion	At 4 Weeks
Absent or mild (score 0–3)	24 (20.3)	85 (72.0)
Moderate (score 4–6)	57 (48.3)	25 (21.2)
Severe (score 7–10)	37 (31.4)	8 (6.8)

IBS-SSS, IBS Symptom Severity Scale. Intragroup test of symmetric distribution: *p* < 0.0001.

## Data Availability

Data that supported the findings of this study are available on request from the corresponding author.
